# Comparison of Response to Definitive Radiotherapy for Localized Prostate Cancer in Black and White Men: A Meta-analysis

**DOI:** 10.1001/jamanetworkopen.2021.39769

**Published:** 2021-12-29

**Authors:** Ting Martin Ma, Tahmineh Romero, Nicholas G. Nickols, Matthew B. Rettig, Isla P. Garraway, Mack Roach, Jeff M. Michalski, Thomas M. Pisansky, W. Robert Lee, Christopher U. Jones, Seth A. Rosenthal, Chenyang Wang, Holly Hartman, Paul L. Nguyen, Felix Y. Feng, Paul C. Boutros, Christopher Saigal, Karim Chamie, William C. Jackson, Todd M. Morgan, Rohit Mehra, Simpa S. Salami, Randy Vince, Edward M. Schaeffer, Brandon A. Mahal, Robert T. Dess, Michael L. Steinberg, David Elashoff, Howard M. Sandler, Daniel E. Spratt, Amar U. Kishan

**Affiliations:** 1 Department of Radiation Oncology, University of California, Los Angeles (UCLA); 2 Statistics Core, David Geffen School of Medicine, UCLA; 3 Department of Radiation Oncology, Veterans Affairs Greater Los Angeles Healthcare System, Los Angeles, California; 4 Division of Hematology and Oncology, David Geffen School of Medicine, UCLA; 5 Division of Hematology and Oncology, Veterans Affairs Greater Los Angeles Healthcare System, Los Angeles, California; 6 Department of Urology, UCLA; 7 Jonsson Comprehensive Cancer Center, David Geffen School of Medicine, UCLA; 8 Division of Urology, Greater Los Angeles Veterans Affairs Healthcare System, Los Angeles, California; 9 Department of Radiation Oncology, Helen Diller Comprehensive Cancer Center, University of California, San Francisco; 10 Washington University School of Medicine in St Louis, St Louis, Missouri; 11 Department of Radiation Oncology, Mayo Clinic, Rochester, Minnesota; 12 Department of Radiation Oncology, Duke University School of Medicine, Durham, North Carolina; 13 Sutter Medical Group and Sutter Cancer Centers, Roseville, California; 14 Department of Radiation Oncology, Division of Radiation Oncology, University of Texas MD Anderson Cancer Center, Houston; 15 Department of Radiation Oncology, University of Michigan, Ann Arbor; 16 Department of Radiation Oncology, Brigham and Women’s Hospital/Dana Farber Cancer Institute, Harvard Medical School, Boston, Massachusetts; 17 Department of Human Genetics, UCLA; 18 Department of Urology, University of Michigan, Ann Arbor; 19 Department of Pathology, University of Michigan, Ann Arbor; 20 Department of Urology, Northwestern University Feinberg School of Medicine, Chicago, Illinois; 21 Department of Radiation Oncology, Cedars-Sinai Medical Center, Los Angeles, California; 22 Department of Radiation Oncology, University Hospitals Seidman Cancer Center, Cleveland Medical Center, Cleveland, Ohio

## Abstract

**Importance:**

Black men have a 2-fold increased risk of dying from prostate cancer compared with White men. However, race-specific differences in response to initial treatment remain unknown.

**Objective:**

To compare overall and treatment-specific outcomes of Black and White men with localized prostate cancer receiving definitive radiotherapy (RT).

**Data Sources:**

A systematic search was performed of relevant published randomized clinical trials conducted by the NRG Oncology/Radiation Therapy Oncology Group between January 1, 1990, and December 31, 2010. This meta-analysis was performed from July 1, 2019, to July 1, 2021.

**Study Selection:**

Randomized clinical trials of definitive RT for patients with localized prostate cancer comprising a substantial number of Black men (self-identified race) enrolled that reported on treatment-specific and overall outcomes.

**Data Extraction and Synthesis:**

Individual patient data were obtained from 7 NRG Oncology/Radiation Therapy Oncology Group randomized clinical trials evaluating definitive RT with or without short- or long-term androgen deprivation therapy. Unadjusted Fine-Gray competing risk models, with death as a competing risk, were developed to evaluate the cumulative incidences of end points. Cox proportional hazards models were used to evaluate differences in all-cause mortality and the composite outcome of distant metastasis (DM) or death. The Preferred Reporting Items for Systematic Reviews and Meta-Analyses (PRISMA) guideline was followed.

**Main Outcomes and Measures:**

Subdistribution hazard ratios (sHRs) of biochemical recurrence (BCR), DM, and prostate cancer-specific mortality (PCSM).

**Results:**

A total of 8814 patients (1630 [18.5%] Black and 7184 [81.5%] White) were included; mean (SD) age was 69.1 (6.8) years. Median follow-up was 10.6 (IQR, 8.0-17.8) years for surviving patients. At enrollment, Black men were more likely to have high-risk disease features. However, even without adjustment, Black men were less likely to experience BCR (sHR, 0.88; 95% CI, 0.58-0.91), DM (sHR, 0.72; 95% CI, 0.58-0.91), or PCSM (sHR, 0.72; 95% CI, 0.54-0.97). No significant differences in all-cause mortality were identified (HR, 0.99; 95% CI, 0.92-1.07). Upon adjustment, Black race remained significantly associated with improved BCR (adjusted sHR, 0.79; 95% CI, 0.72-0.88; *P* < .001), DM (adjusted sHR, 0.69; 95% CI, 0.55-0.87; *P* = .002), and PCSM (adjusted sHR, 0.68; 95% CI, 0.50-0.93; *P* = .01).

**Conclusions and Relevance:**

The findings of this meta-analysis suggest that Black men enrolled in randomized clinical trials present with more aggressive disease but have better BCR, DM, and PCSM with definitive RT compared with White men, suggesting that other determinants of outcome, such as access to care, are important factors of achieving racial equity.

## Key Points

**Question:**

Is there a difference in outcomes between Black and White men with localized prostate cancer receiving definitive radiotherapy (RT)?

**Findings:**

In this meta-analysis that included 8814 patients treated with definitive RT enrolled in 7 randomized clinical trials, Black men were significantly less likely to experience a biochemical recurrence, distant metastasis, and prostate cancer–specific mortality event than White men.

**Meaning:**

The findings of this meta-analysis noted that Black men enrolled in randomized clinical trials presented with more aggressive disease features but had better treatment and disease-specific outcomes with RT-based therapy compared with White men, suggesting other important factors associated with outcome, such as access to care, as sources of disparity.

## Teaser

This meta-analysis assesses treatment-specific outcomes associated with receipt of definitive radiotherapy in Black and White men with localized prostate cancer.

## Introduction

Black men are more likely to be diagnosed with prostate cancer and are more likely to die from the disease compared with White men.^1^ Evidence suggests that a large proportion of these differences may be attributable to socioeconomic factors and/or disparities in guideline-concordant care.^2,3,4^ Data also suggest that biological differences may exist that could help explain in part the observed population-based disparities in prostate cancer outcomes.^5,6,7^

In the context of metastatic disease, data suggest improved efficacy of abiraterone,^8^ docetaxel,^9^ and sipuleucel-T ^10^ in Black compared with non-Black men. Additional studies suggest prostate cancer–specific mortality (PCSM) outcomes are similar for patients receiving definitive therapy for localized disease, provided equal access to care, and receiving standardized treatments.^2^ However, the end point of PCSM, albeit important, is the culmination of often many years of multiple salvage therapies and does not intrinsically capture the initial responsiveness to primary therapy. To our knowledge, no large-scale study has been conducted to examine race and the early metrics of response to treatment, including biochemical recurrence (BCR) or the development of distant metastasis (DM) in men with localized prostate cancer; thus, it is unknown whether there is an initial differential response to treatment by race. Because most patients with prostate cancer present with localized disease, understanding potential differences in the initial response to therapy is necessary to identify potential factors and/or mitigators of disparities in prostate cancer care.

To elucidate associations between race and both early (ie, BCR and DM) and late (ie, PCSM and all-cause mortality [ACM]) outcomes of treatment efficacy among men with localized prostate cancer, we performed the largest individual patient data meta-analysis to date of men with localized prostate cancer enrolled in 7 randomized trials using definitive RT with varying schedules of androgen deprivation therapy (ADT).

## Methods

### Patients and Trial Inclusion

We performed a systematic literature search to identify relevant randomized clinical trials run by the NRG Oncology/Radiation Therapy Oncology Group (RTOG) from January 1, 1990, to December 31, 2010, which has historically enrolled a substantial number of Black men in its trials.^11,12^ This systematic review and meta-analysis was performed from July 1, 2019, to July 1, 2021. Seven trials for which individual patient-level data were available were identified (eFigure 1 and eTable 1 in the Supplement). Data sharing applications were submitted to NRG Oncology to obtain individual patient-level data for patients enrolled in RTOG protocols 9202,^13^ 9408,^14^ 9413,^15^ 9902,^16^ 9910,^17^ 0126,^18^ and 0415.^19^ Specific information on inclusion criteria and treatment details is presented in eTable 1 in the Supplement. Patients who had node-positive disease (clinically or via pathologic sampling) were excluded. Data were extracted for men who self-identified as Black or White race and reviewed by ^2^ of us (T.R. and A.U.K.). The arms of each trial were merged into 1 of 4 larger groups (ie, treatment strategies): RT alone, RT with short-term ADT, RT with long-term ADT, and high-dose RT (eTable 2 in the Supplement). RT doses higher than 74 Gy were considered high dose (presuming an α/β ratio of 3.0 to convert hypofractionated schedules). The duration of short-term ADT was 4 months (except for RTOG 9910, in which the arm with 9 months of ADT was included as it was not oncologically different from the 4-month ADT arm) while that of long-term ADT was 24 to 28 months. The study was approved by the University of California Los Angeles Institutional Review Board. This study followed the Preferred Reporting Items for Systematic Reviews and Meta-analyses (PRISMA) reporting guideline.

### Study End Points

The primary outcomes of interest for the current analysis were BCR, DM, and PCSM. For BCR, in all trials (except RTOG 9902), the Phoenix definition (increase of prostate-specific antigen [PSA] level by ≥2 ng/mL [1:1 conversion to micrograms per liter] above the nadir) was used. For RTOG 9902, the ASTRO definition (3 consecutive PSA increases after a nadir) was used. All-cause mortality was considered a secondary end point because data regarding comorbidity status were not available, and comorbidity status has been shown to limit estimation of ACM, both for prostate cancer in general^20^ and when investigating associations between race and outcome specifically.^2^ A composite outcome, defined as death or DM, was also explored as the inverse of metastasis-free survival—a validated surrogate marker for overall survival in patients receiving definitive RT for prostate cancer.^21^ Time to event was defined as per each study (generally, from time of randomization to the end point in question).

### Statistical Analysis

Differences in age and initial PSA level between races were evaluated with a Wilcoxon rank sum test, and differences in categorical variables (including PSA level with cut points, performance status, National Comprehensive Cancer Network (NCCN) risk group, Gleason score [higher scores indicate greater risk], and cT category) were compared using the Fisher exact test. Cumulative incidences of BCR, DM, PCSM, and other-cause mortality for each trial were estimated with a competing risk method. Cumulative incidences of death or DM and of ACM were estimated for each trial with Kaplan-Meier methods. Each trial-level estimate was then pooled to provide an estimate for the entire cohort using a meta-analysis approach with random effects. We also developed cumulative incidence and survival curves that were weighted for the inverse probability of being enrolled in a given trial. Weights were determined based on a multinomial logistic regression with trial as the outcome and age, initial PSA level, Gleason score, T category, and treatment strategy as independent covariates. To evaluate associations between race and BCR, DM, and PCSM, a network meta-analysis was performed. First, the trial-specific subdistribution hazard ratios (sHRs) for the associations between race and BCR, DM, and PCSM in the presence of competing risks were estimated using the multivariable Fine-Gray method with age at treatment, ln(initial PSA level), treatment strategy, T category, and Gleason score as independent covariates. Death from any cause was considered a competing risk for BCR and DM, and other-cause mortality was considered a competing risk for PCSM. Other-cause mortality was modeled similarly, with PCSM as the competing risk event. Next, trial-specific estimates were combined using traditional meta-analysis methods with random effects to obtain a pooled overall estimate.

The same approach was used to conduct a 2-step random effect meta-analysis to estimate the unadjusted association between race and each end point within predefined subgroups. The categories were defined as age (≤65 vs >65 years), NCCN risk grouping,^22^ PSA level (<10, 10-20, >20 ng/mL), Gleason score (6, 7, 8-10), T category (T1-2 vs T3-4), and treatment strategy (RT alone, RT with short-term ADT, RT with long-term ADT, and high-dose RT alone). To adjust for multiple comparisons when estimating within categories (HRs and sHRs), we report *q* values that were adjusted for false discovery rates. *P* values were 2-tailed, and statistical significance was set at *P* = .05. All statistical analyses were conducted using SAS, version 9.4 (SAS Institute Inc) and Packages metafor and netmeta (Network Meta-Analysis using Frequentist Methods)^23^ in R, version 3.3.1.^24^

## Results

### Patient and Trial Characteristics

Overall, 8814 patients (Black, 1630 [18.5%]; White, 7184 [81.5%]) were identified. The mean (SD) age overall was 69.1 (6.8) years (Black, 67.1 [7.3] years; White, 69.6 [6.6] years). The cohort comprised primarily patients with NCCN risk levels of low (1748 [19.8%]) and intermediate (4263 [48.4%]); the remaining patients had high-risk disease (2803 [31.8%]). Risk groups were defined by the NCCN^22^ as low risk (cT1-T2a, Gleason score ≤6, and PSA level <10 ng/mL), intermediate risk (cT2b-T2c, Gleason score 7, or PSA level 10-20 ng/mL, and high risk (cT3a or Gleason score 8-10 or PSA level >20 ng/mL). Patient characteristics are presented in Table 1. Median follow-up was 10.6 (IQR, 8.0-17.8) years for living patients using the inverse Kaplan-Meier method. On average, 19.5% of patients in any of the 7 evaluated studies were of Black race, with RTOG 0126 having the lowest percentage of Black patients (188 of 1440 [13.1%]), and RTOG 9902 having the highest (102 of 367 [27.8%]).

**Table 1.** Baseline Clinical and Demographic Characteristics

Variable	No. (%)		*P* value^a^
	Black race (n = 1630)	White race (n = 7184)	
Age, y			
Mean (SD)	67.1 (7.3)	69.6 (6.6)	<.001
Median (IQR)	68 (62-73)	71 (66-74)	<.001
NCCN risk			
Low	325 (19.9)	1423 (19.8)	<.001
Intermediate	683 (41.9)	3580 (49.8)	
High	622 (38.2)	2181 (30.4)	
PSA level, ng/mL^b^			
Mean (SD)	16.2 (16.4)	12 (12.1)	<.001
Median (IQR)	10.3 (6.2-19.1)	8.4 (5.7-13.2)	<.001
PSA level, ng/mL^b^			
<10	704/1527 (46.1)	3631/6327 (57.4)	<.001
10-20	490/1527 (32.1)	2005/6327 (31.7)	
>20	333/1527 (21.8)	691/6327 (10.9)	
Gleason score^c^			
6	733/1608 (45.6)	2975/7086 (42.0)	<.001
7	613/1608 (38.1)	3114/7086 (43.9)	
8	173/1608 (10.8)	599/7086 (8.5)	
9	80/1608 (5.0)	345/7086 (4.9)	
10	9/1608 (0.6)	53/7086 (0.7)	
T category			
1	772/1528 (50.5)	2857/6919 (41.3)	<.001
2	497/1528 (32.5)	2766/6919 (40.0)	
3	206/1528 (13.5)	968/6919 (14.0)	
4	53/1528 (3.5)	328/6919 (4.7)	
Trial			
RTOG 9202	186 (11.4)	1228 (17.1)	<.001
RTOG 9408	394 (24.2)	1497 (20.8)	
RTOG 9413	322 (19.8)	881 (12.3)	
RTOG 9902	102 (6.3)	265 (3.7)	
RTOG 9910	246 (15.1)	1190 (16.6)	
RTOG 0126	188 (11.5)	1252 (17.4)	
RTOG 0415	192 (11.8)	871 (12.1)	

Abbreviations: NCCN, National Comprehensive Cancer Network; PSA, prostate-specific antigen.

SI conversion of PSA levels to micrograms per liter is 1:1.

^a^ *P* value was calculated excluding patients in the unknown category for each variable.

^b^ Individual PSA values not known for RTOG 9202, because only PSA levels dichotomized to less than or equal to 30 vs greater than 30 ng/mL were provided.

^c^ Higher scores indicate greater risk.

Overall, Black men presented at a significantly younger age (median [IQR], 68 [62-73] vs 71 [66-74] years; *P* < .001). They were also significantly more likely to present with high-risk disease (622 [38.2%] vs 2181 [30.4%]; *P* < .001), higher PSA levels (median [IQR], 10.3 [6.2-19.1] vs 8.4 [5.7-13.2] ng/mL; *P* < .001), and Gleason scores of 8 to 10 (262 of 1608 [16.3%] vs 997 of 7086 [14.1%]; *P* = .03). However, there was no difference in the proportion of patients with cT3-4 disease (259 [17.0%] vs 1296 [18.7%]; *P* = .10), although Black men were more likely to have cT1 disease (772 [50.5%] vs 2857 [41.3%]; *P* < .001).

### Cumulative Incidences of End Points

**Figure 1.**
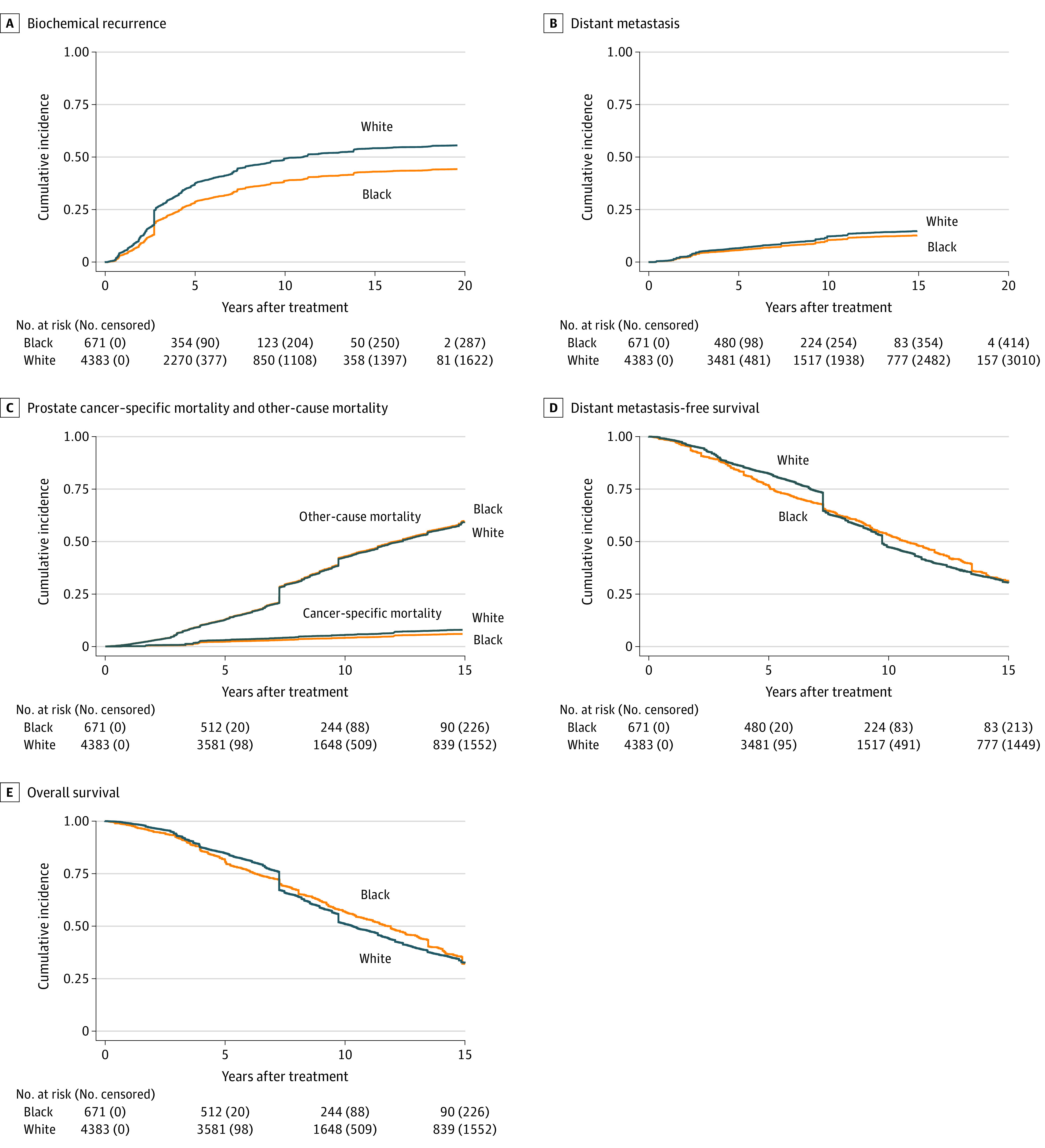
Cumulative Incidence and Survival Curves Weighted for the Inverse Probability of Trial Enrollment Cumulative incidence curves for biochemical recurrence (A), distant metastasis (B), prostate cancer–specific mortality and other-cause mortality (C), distant metastasis-free survival (D), and overall survival (E). All curves were weighted for the inverse probability of enrollment on a given trial. Weights were determined based on a multinomial logistic regression with trial as the outcome and age, prostate-specific antigen level, Gleason score, T category, and treatment strategy as independent covariates.

Individual trial crude event rates within the Black and White subgroups are reported in eTable 3 in the Supplement. Compared with White men, Black men had lower absolute unadjusted 10-year cumulative incidence rates of BCR (40.5% vs 44.6%; *P* = .006), DM (8.4% vs 11.6%; *P* = .005), and PCSM (4.5% vs 6.4%; *P* = .03). Ten-year rates of ACM and death or DM were similar (ACM: 39.8% vs 41.2%; log-rank test, *P* = .43; death or DM: 41.5% vs 43.6%; log-rank test, *P* = .40). Other-cause mortality was also similar (10-year rates of 37.2% vs 36.6%; *P* = .50). Proportions of death attributable to PCSM vs other-cause mortality overall and based on age and NCCN risk group are shown in eFigure 2 in the Supplement, with additional information on breakdown by other prespecified subgroups shown in eFigure 3 in the Supplement. A lower percentage of mortality events were due to PCSM rather than other-cause mortality overall (50 of 773 [6.5%] vs 368 of 3617 [10.2%]) and among men younger than 65 years (17 of 225 [7.6%] vs 93 of 624 [14.9%]) and aged 65 years or older (33 of 548 [6.0%] vs 275 of 2993 [9.2%]) as well as men with high-risk disease (24 of 374 [6.4%] vs 188 of 1480 [12.7%]). To account for ecological biases and include time to event considerations, we also developed cumulative incidence and survival curves that were weighted for the inverse probability of being enrolled in a given trial (Figure 1).

### Competing Risk and Cox Proportional Hazards Models

**Figure 2.**
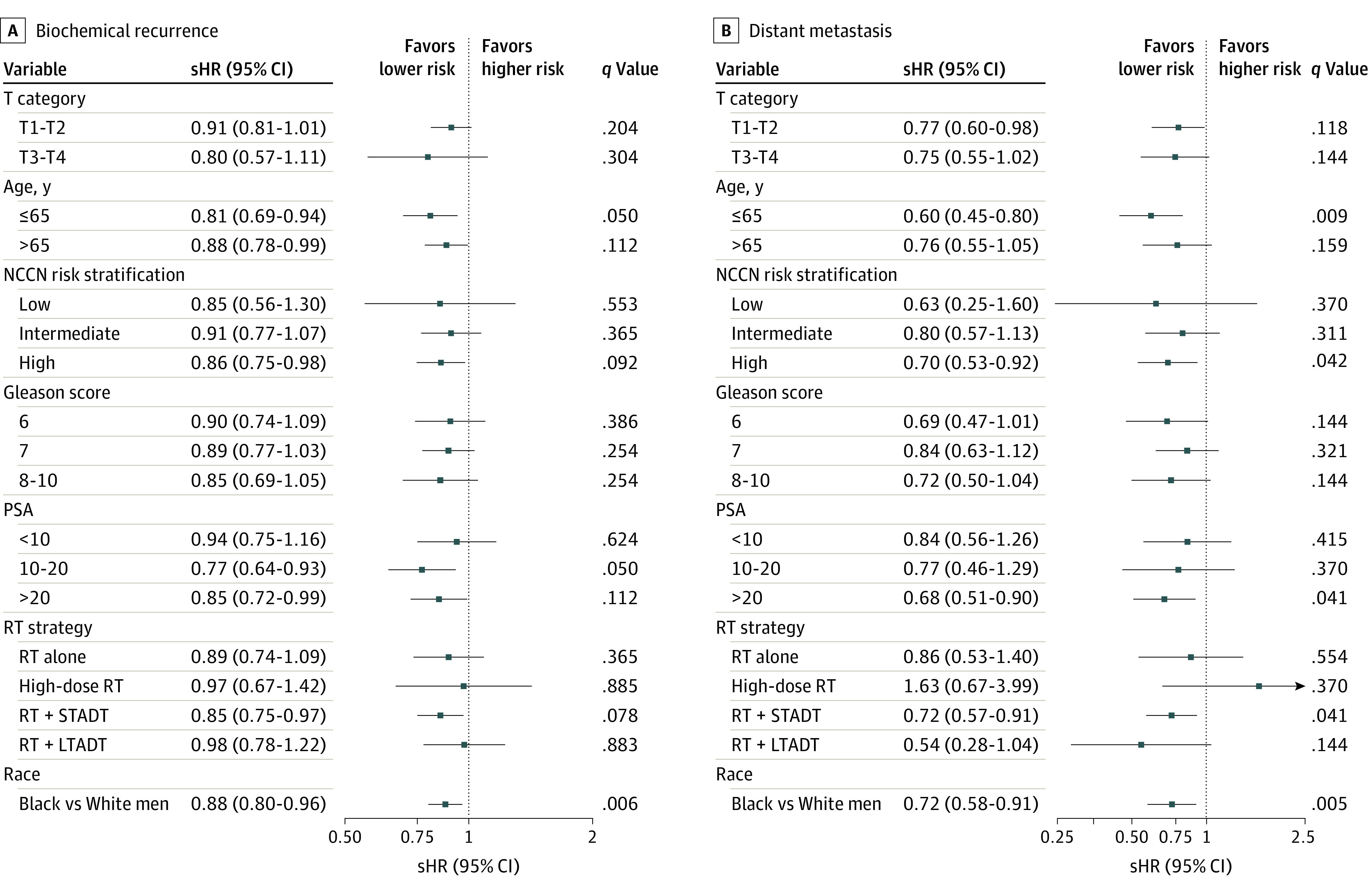
Estimates of Association Between Race and Biochemical Recurrence and Distant Metastasis Associations between race and biochemical recurrence (A) and distant metastasis (B) were modeled with the unadjusted Fine-Gray method yielding subdistribution hazard ratio (sHR) method. Trial-specific estimates were generated and then combined with a 2-step meta-analysis method with random effects to obtain overall estimates. LTADT indicates long-term androgen deprivation therapy; NCCN, National Comprehensive Cancer Network; PSA, prostate-specific antigen; RT, radiotherapy; sHR, subdistribution hazard ratio; and STADT, short-term androgen deprivation therapy. SI conversion of PSA levels to micrograms per liter is 1:1.

**Figure 3.**
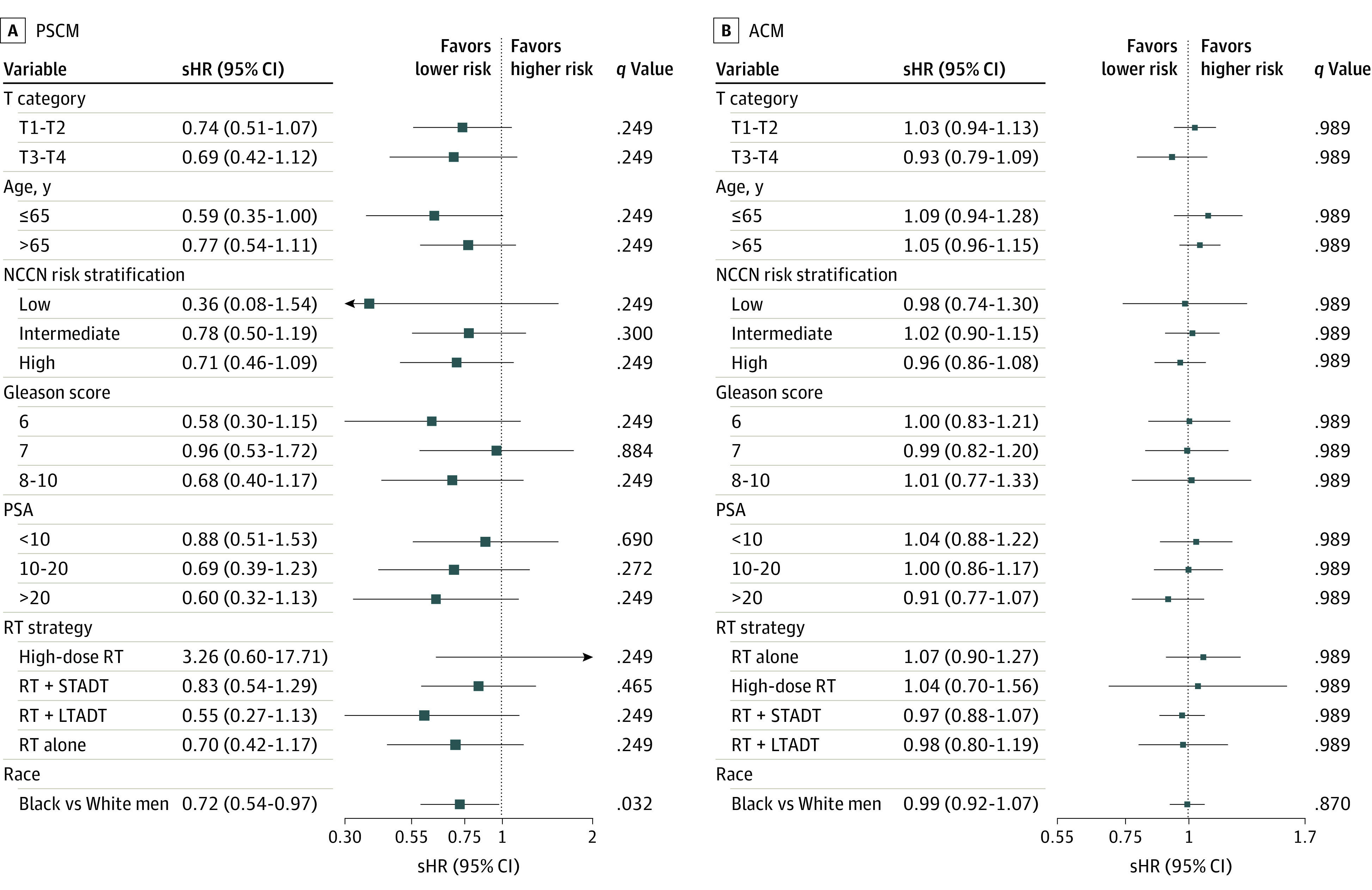
Estimates of Association Between Race and Prostate Cancer–Specific Mortality (PCSM) and All-Cause Mortality (ACM) Associations between race and prostate cancer–specific mortality (A) and all-cause mortality (B) were modeled with the unadjusted Fine-Gray method yielding subdistribution hazard ratio (sHR) and Cox proportional hazards models yielding hazard ratios (HRs), respectively. Trial-specific estimates were generated and then combined with a 2-step meta-analysis method with random effects to obtain overall estimates. LTADT indicates long-term androgen deprivation therapy; NCCN, National Comprehensive Cancer Network; PSA, prostate-specific antigen; RT, radiotherapy; sHR, subdistribution hazard ratio; and STADT, short-term androgen deprivation therapy. SI conversion of PSA levels to micrograms per liter is 1:1.

The forest plots depicting the results of our 2-step unadjusted meta-analysis evaluating BCR, DM, PCSM, and ACM are shown in Figure 2 and Figure 3. Overall, Black race was associated with a lower risk of BCR (sHR, 0.88; 95% CI, 0.80-0.96; *P* = .006), DM (sHR, 0.72; 95% CI, 0.58-0.91; *P* = .005), and PCSM (sHR, 0.72; 95% CI, 0.54-0.97; *P* = .03). There was no significant difference in time to ACM (HR, 0.99; 95% CI, 0.92-1.07; *P* = .87). Similarly, there was no significant difference in time to other-cause mortality (sHR, 1.03; 95% CI, 0.95-1.12; *P* = .50), and DM or death (HR, 1.00; 95% CI, 0.92-1.08; *P* = .91) (eFigure 4 in the Supplement). When examined within predefined strata, Black race was similarly associated with a lower risk of BCR, DM, and PCSM among men aged 65 years or younger. Black race was also associated with a lower risk of BCR and DM among men with high-risk disease, PSA level greater than 20 ng/mL, and men receiving RT with short-term ADT. We also evaluated the association between race and BCR, DM, and PCSM with a network meta-analysis while adjusting for age, initial PSA level, T category, Gleason score, and treatment strategy (Table 2). Black race was significantly associated with improved BCR (adjusted sHR, 0.79; 95% CI, 0.72-0.88; *P* < .001), DM (adjusted sHR, 0.69; 95% CI, 0.55-0.87; *P* = .002), and PCSM (adjusted sHR, 0.68; 95% CI, 0.5-0.93; *P* = .01).

**Table 2.** Multivariable Competing Risk Analysis for Factors Associated With Treatment Outcome

Characteristic	BCR		DM		PCSM	
	sHR (95% CI)	*P* value	sHR (95% CI)	*P* value	sHR (95% CI)	*P* value
Race (Black vs White)	0.79 (0.72-0.88)	<.001	0.69 (0.55-0.87)	.002	0.68 (0.50-0.93)	.01
Treatment strategy						
RT + STADT vs RT alone	0.63 (0.54-0.73)	<.001	0.64 (0.46-0.88)	.006	0.55 (0.40-0.75)	<.001
RT+ LTADT vs RT alone	0.37 (0.30-0.46)	<.001	0.41 (0.28-0.62)	<.001	0.38 (0.25-0.58)	<.001
High dose RT vs RT alone	0.60 (0.51-0.72)	<.001	0.64 (0.40-1.00)	.05	0.37 (0.15-0.92)	.03
RT+ LTADT vs RT + STADT	0.59 (0.50-0.69)	<.001	0.65 (0.51-0.83)	<.001	0.70 (0.53-0.92)	.01
High dose RT vs RT + STADT	0.96 (0.77-1.20)	.74	1.00 (0.57-1.75)	.10	0.68 (0.26-1.78)	.43
High dose RT vs RT + LTADT	1.64 (1.25-2.15)	<.001	1.55 (0.84-2.84)	.16	0.98 (0.36-2.66)	.97
Age (1-y increase)	0.98 (0.97-0.99)	<.001	0.97 (0.96-0.99)	<.001	0.98 (0.95-1.01)	.18
Ln(iPSA) (1-U increase)	1.60 (1.41-1.80)	<.001	1.36 (1.23-1.51)	<.001	1.80 (1.06-3.04)	.03
T category (1-U increase)	1.12 (1.05-1.20)	.001	1.23 (1.11-1.37)	<.001	1.60 (0.88-2.90)	.12
Gleason score (1-U increase)	1.22 (1.14-1.31)	<.001	1.48 (1.27-1.74)	<.001	1.55 (1.41-1.70)	<.001

Abbreviations: BCR, biochemical recurrence; DM, distant metastasis; LTADT, long-term androgen deprivation therapy; PCSM, prostate cancer-specific mortality; RT, radiotherapy; STADT, short-term androgen deprivation therapy; sHR, subdistribution hazard ratio.

## Discussion

In this individual patient data meta-analysis of 7 randomized clinical trials, Black men were significantly more likely to have high-risk disease and a younger age at the time of treatment, yet had lower BCR, DM, and PCSM rates compared with White men, even without adjustment. In an adjusted network meta-analysis that accounted for age, initial PSA level, T category, Gleason score, and treatment strategy, race remained significantly associated with improved BCR, DM, and PCSM outcomes. No significant differences were found with respect to ACM or the composite outcome DM or death, and most mortality events in either Black or White men were other-cause mortality events. The fact that Black men had improved early and late disease outcomes compared with White men is a novel and unexpected result, suggesting that Black men may have an improved response to their initial treatment.

A 2019 meta-analysis focusing on men with advanced disease reported a significant increase in overall survival in Black vs White men with metastatic castrate-resistant prostate cancer treated with docetaxel in phase 3 clinical trials.^9^ Other data suggest a similar increased efficacy of abiraterone^8^ and sipeulecel-T.^10^ An earlier report, predominantly assessing men with prostate cancer diagnosed between 1980 and 1990, found that Black men presenting with metastatic prostate cancer had improved survival compared with White men.^25^ To our knowledge, whether a parallel association between Black race and improved early disease outcomes (ie, BCR and DM) might be present among men presenting with localized disease—comprising most men with newly diagnosed prostate cancer—has not been extensively studied. These earlier end points are important given the prolonged natural history of localized prostate cancer both in general^26,27^ and after BCR,^28,29^ as well as the availability of effective treatments for patients with DM.^30^ This study provides, to our knowledge, the most comprehensive analysis of the association between race and multiple end points in patients with localized prostate cancer.

The results should be contextualized with other studies that have focused on potential associations between race and outcomes in men with localized prostate cancer. A recent meta-analysis, which included 4 of the 7 randomized clinical trials we used, reported that Black race was not associated with worse PCSM outcome.^2^ In that study, analyses were adjusted to account for imbalances in age and risk features (including risk group itself) to underscore the disparities in access to care and uncontrolled treatment selection on PCSM outcomes. McKay et al^31^ recently reported the outcomes in patients with nonmetastatic prostate cancer treated with definitive radiotherapy at 152 centers within the Veterans Health Administration and found that Black race was associated with a decreased risk of PCSM and ACM. In the present report, we broadened the analysis to a larger cohort of men (including 44% more Black men) and specifically interrogated not only PCSM, but earlier disease outcomes, such as BCR and DM. In our main 2-step meta-analysis approach, we did not perform any adjustments, although differences in important prognostic variables, such as NCCN risk stratum and Gleason score, were present. We did not adjust the analyses because other important variables, such as percentage of positive biopsy cores, primary Gleason score, and performance status, were not readily available, we believed it was most appropriate to evaluate unadjusted competing risks and incidence rates. However, we performed an adjusted network meta-analysis that supported our finding that race appeared to be independently associated with improved BCR, DM, and PCSM.

These results provide high-level evidence to question the belief that prostate cancer among Black men necessarily portends a worse prognosis compared with White men. This belief may be a factor in differences in the approach to cancer therapy, thereby leading to the use of more aggressive treatments than might be necessary, which carry greater risks of decreasing the quality of life and distracting attention from other important factors associated with outcome and sources of disparity, such as access to care.^32,33^ The findings between race and outcome in our analysis were derived from patient groups that not only had access to enrollment but were enrolled in randomized clinical trials, with all patients (Black and White) receiving the same treatment. There is an important distinction between access to trials and enrollment as Black men have been reported to be notably less willing to discuss trials than White men owing to medical mistrust.^34^ In the general population, such equity in access to care and receipt of treatment are not realized, therefore leading to population-level disparities in outcome. Engaging Black men and increasing the representation of the Black population in various cancer prevention and treatment studies is warranted and can be facilitated by connecting with community stakeholders and identifying study champions.^35^ Moreover, most death events in both Black and White men are due to diseases other than prostate cancer, underscoring the importance of overall health in men with prostate cancer. Nononcologic care is needed, and disparities in overall health care access and receipt can also be factors associated with survival outcomes on a population level. However, these results do not suggest that there are no biological differences that might be associated with differences in prostate cancer incidence between racial groups. It is possible that the association with differential treatment response might be, at least in part, explained by differences in underlying biologic factors. Studies have reported distinct characteristics of prostate cancer in Black and White men at the genetic,^36,37,38^ epigenetic,^39^ and immunological level.^40^ These differences may have contributed to improved efficacy of multiple lines of systemic therapy in Black men compared with non-Black men with locally advanced or metastatic disease.^10^

### Limitations

This study has limitations. First, none of the trials included in this analysis was designed to investigate associations between race and outcome of the trial intervention. Therefore, these comparisons are post hoc and susceptible to residual confounding beyond what would be expected for optimal prespecified subgroup analyses.^41^ Second, race was defined on the basis of self-identification and is a sociopolitical construct that may not intrinsically capture biologic factors.^42^ Ancestry may be a more appropriate metric for capturing biological phenomena and increasing our understanding of health disparities, but has not been historically abstracted for clinical trials. Third, salvage therapies after BCR or DM were not standardized, potentially leading to secondary confounding of the end points of PCSM and ACM. We addressed this limitation by including these earlier outcomes as end points of interest as well, but it is also possible that intervention after the development of BCR might have had a differential effect on the incidence of DM. Fourth, data for other prognostic variables, such as comorbidity, socioeconomic status, and performance status, were not uniformly available and could not be adjusted for. It is possible that a lack of such information and the inability to adjust thereof precluded us from detecting a significant difference in ACM. Given the known barriers toward enrolling individuals of minority racial and ethnic groups in clinical trials,^43^ the external validity of our results may be different for Black and White men.

## Conclusions

In this meta-analysis, Black men enrolled in randomized clinical trials with long-term follow-up appeared to have higher risk disease features at the time of trial enrollment, but nonetheless had better BCR, DM, and PCSM outcomes with RT-based therapy compared with White men. The findings suggest that Black race may be an independent favorable prognostic variable.

## Notes

Supplement. **eFigure 1.** Preferred Reporting Items for Systematic Reviews and Meta-analyses Flowchart **eFigure 2.** Percentage of All-Cause Mortality Events Attributable to Prostate Cancer–Specific Mortality **eFigure 3.** Percentage of All-Cause Mortality Events Attributable to Prostate Cancer–Specific Mortality in Specific Subgroups **eFigure 4.** Forest Plots of Association Between Race and Other Cause Mortality and Death or Distant Metastasis **eTable 1.** Summary of Trials Included in Network Meta-analysis **eTable 2.** Treatment Strategies as Defined by Individual Trial Treatment Arms **eTable 3.** Crude Event Rates Per Trial, Stratified by Race and Treatment Strategy
