# Supplementary material for: Comparison of Response to Definitive Radiotherapy for Localized Prostate Cancer in Black and White Men: A Meta-analysis
Source: JAMA Netw Open. 2021 Dec 29;4(12):e2139769. doi: 10.1001/jamanetworkopen.2021.39769 (PMC8717118; doi:10.1001/jamanetworkopen.2021.39769)
Supplement: Supplement. — eFigure 1. Preferred Reporting Items for Systematic Reviews and Meta-analyses Flowchart eFigure 2. Percentage of All-Cause Mortality Events Attributable to Prostate Cancer–Specific Mortality eFigure 3. Percentage of All-Cause Mortality Events Attributable to Prostate Cancer–Specific Mortality in Specific Subgroups eFigure 4. Forest Plots of Association Between Race and Other Cause Mortality and Death or Distant Metastasis eTable 1. Summary of Trials Included in Network Meta-analysis eTable 2. Treatment Strategies as Defined by Individual Trial Treatment Arms eTable 3. Crude Event Rates Per Trial, Stratified by Race and Treatment Strategy [file jamanetwopen-e2139769-s001.pdf]

Supplementary Online Content

Ma TM, Romero T, Nickols NG, et al. Comparison of response to definitive radiotherapy for localized prostate cancer in Black and White men: a meta-analysis. *JAMA Netw Open*. 2021;4(12):e2139769. doi:10.1001/jamanetworkopen.2021.39769

eFigure 1. Preferred Reporting Items for Systematic Reviews and Meta-analyses Flowchart

eFigure 2. Percentage of All-Cause Mortality Events Attributable to Prostate Cancer–Specific Mortality

eFigure 3. Percentage of All-Cause Mortality Events Attributable to Prostate Cancer–Specific Mortality in Specific Subgroups

eFigure 4. Forest Plots of Association Between Race and Other Cause Mortality and Death or Distant Metastasis

eTable 1. Summary of Trials Included in Network Meta-analysis

eTable 2. Treatment Strategies as Defined by Individual Trial Treatment Arms

eTable 3. Crude Event Rates Per Trial, Stratified by Race and Treatment Strategy

This supplementary material has been provided by the authors to give readers additional information about their work.

© 2021 Ma TM et al. *JAMA Network Open.*

eFigure 1. Preferred Reporting Items for Systematic Reviews and Meta-Analyses Flowchart

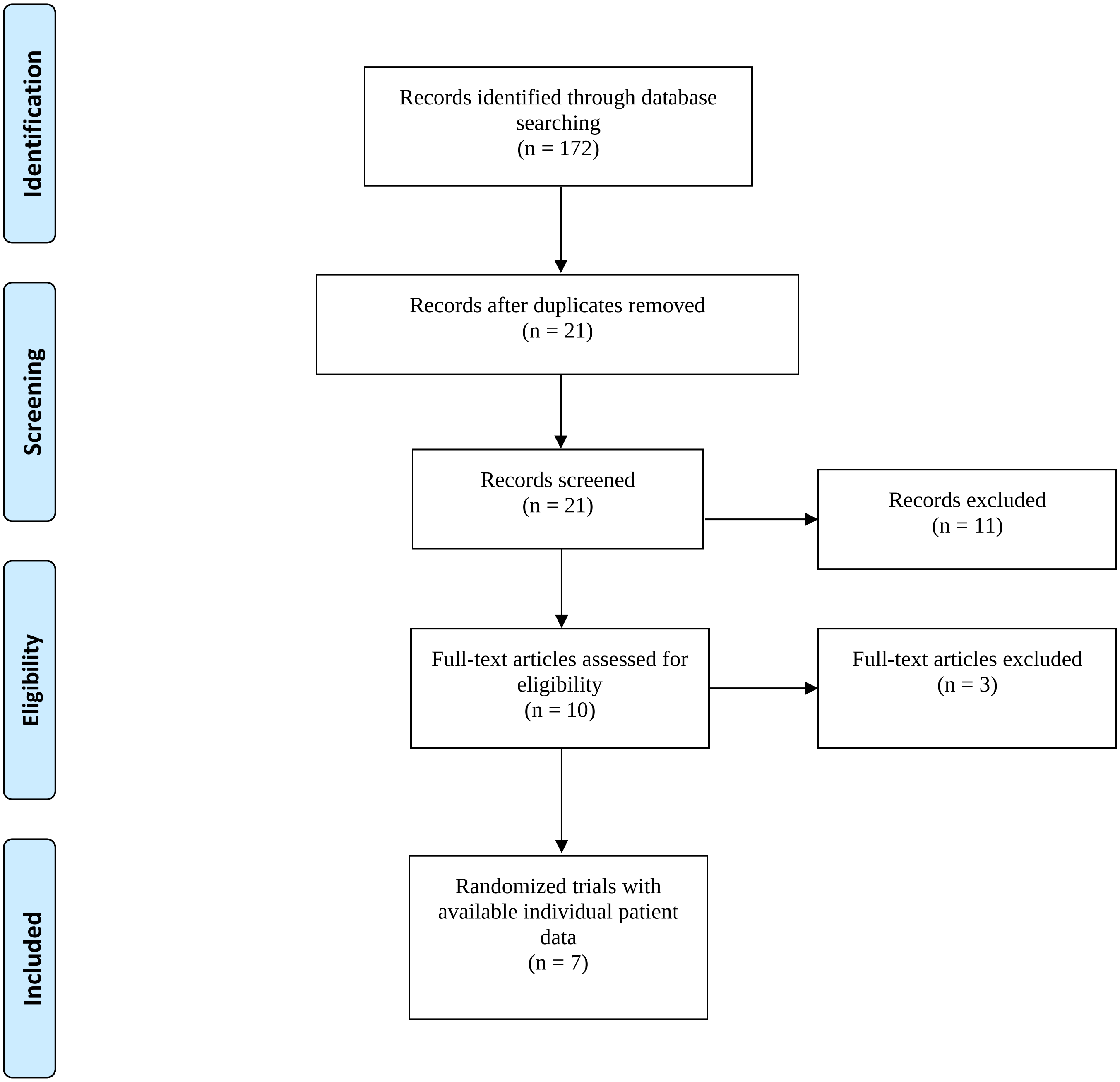

© 2021 Ma TM et al. *JAMA Network Open.*

eFigure 2. Percentage of All-Cause Mortality Events Attributable to Prostate Cancer-Specific Mortality. A lower percentage of mortality events were due to PCSM rather than OCM overall (50/773 [6.5%] vs. 368/3617 [10.2%]), and among men aged <65 (17/225 [7.6%] vs. 93/624 [14.9%]) and ≥65 (33/548 [6.0%] vs. 275/2993 [9.2%]) as well as men with high-risk disease (24/374 [6.4%] vs. 188/1480 [12.7%]).

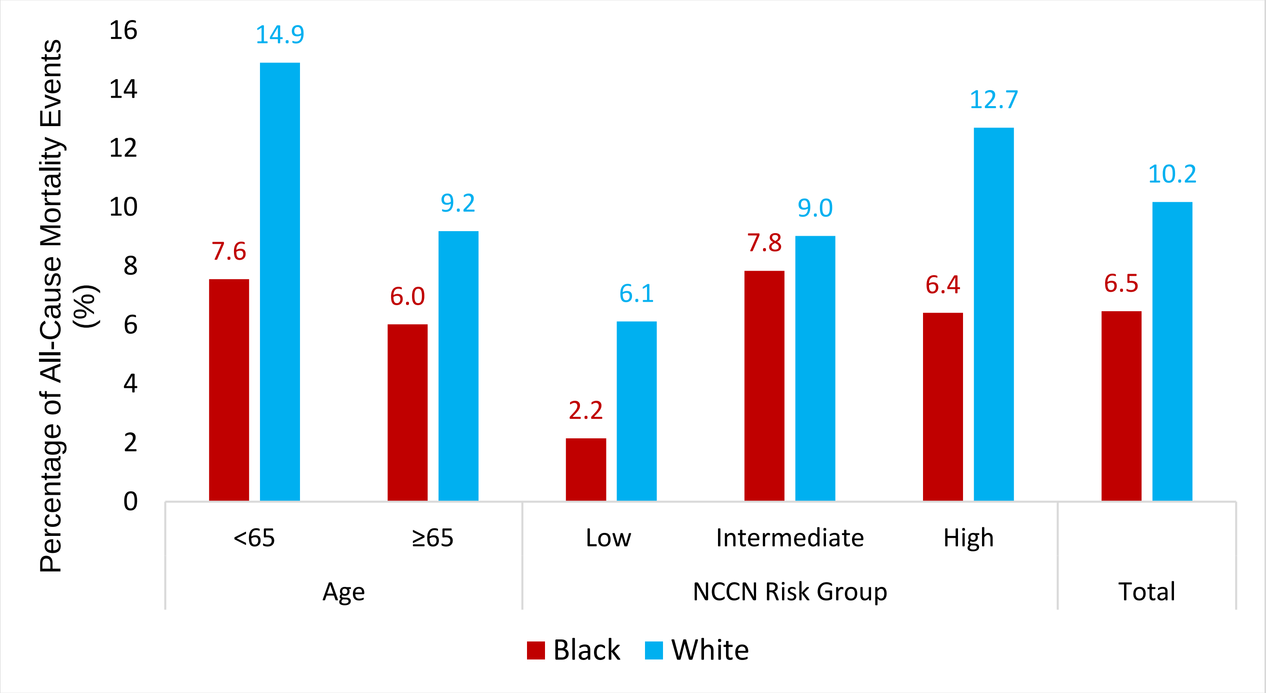

© 2021 Ma TM et al. *JAMA Network Open.*

eFigure 3. Percentage of All-Cause Mortality Events Attributable to Prostate Cancer-Specific Mortality in Specific Subgroups

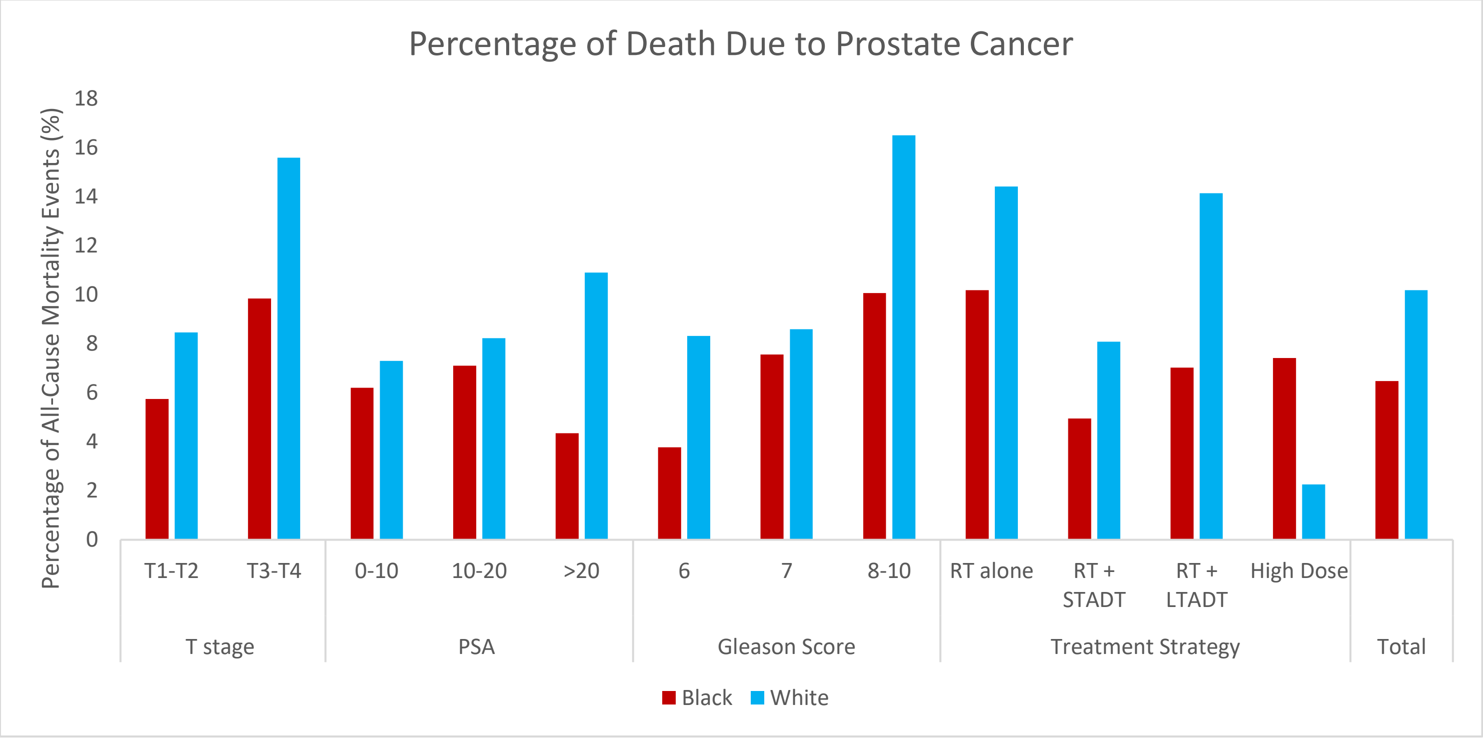

© 2021 Ma TM et al. *JAMA Network Open.*

eFigure 4. Forest Plots of Association Between Race and Other Cause Mortality and Death or Distant Metastasis

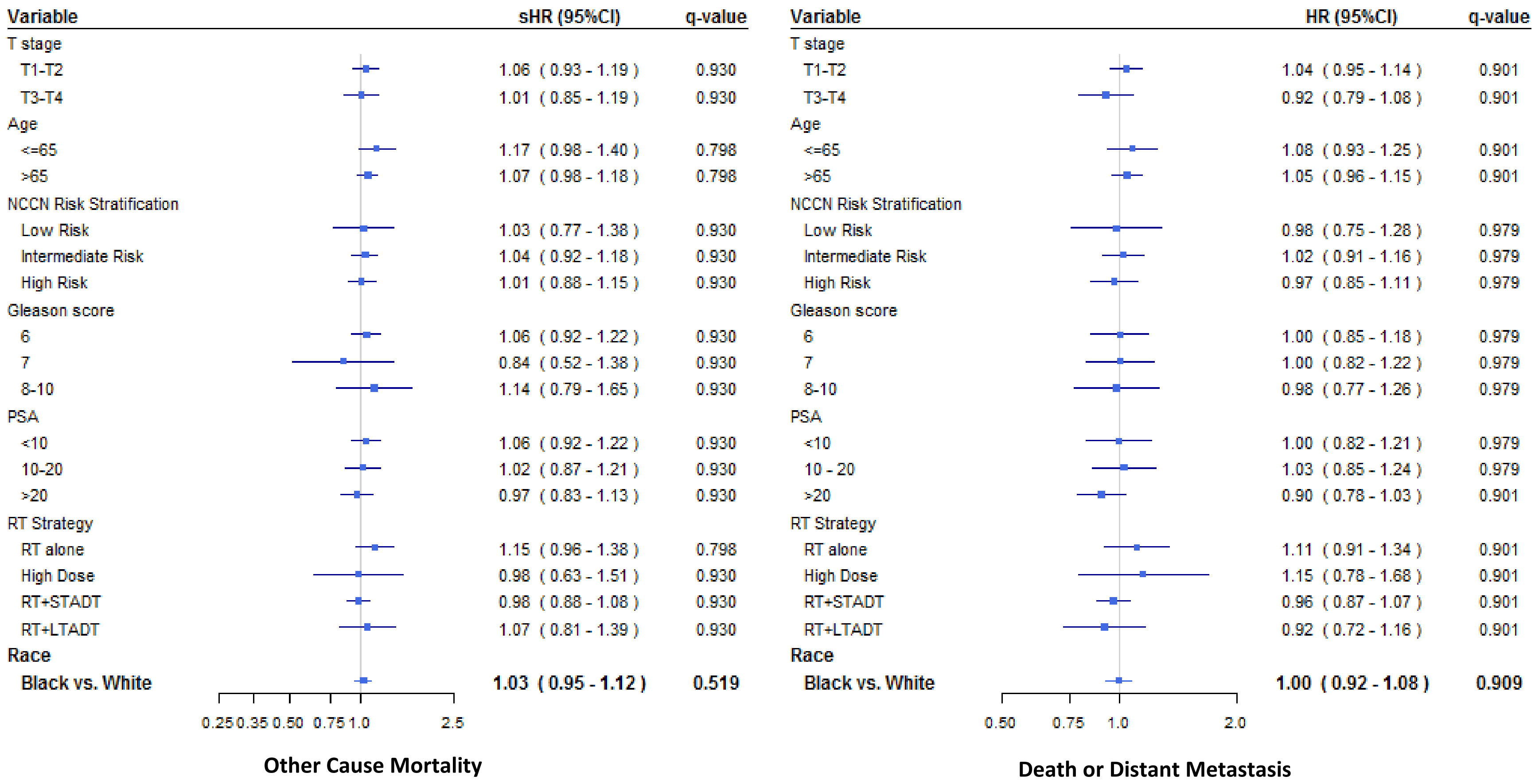

© 2021 Ma TM et al. *JAMA Network Open.*

eTable 1. Summary of Trials Included in Network Meta-Analysis

Trial	Arms	Inclusion*	Primary Endpoint	RT Dose
RTOG 9202 (1992-1995)	RT + 4 mos ADT<br>RT + 28 mos ADT	cT2-4N0-X, PSA<150	Disease-free survival	65-70 Gy to prostate<br>44-46 Gy to pelvis
RTOG 9408 (1994-2001)	RT<br>RT + 4 mos ADT	cT1b-T2b<br>PSA≤20	Overall survival	66.6 Gy to prostate<br>46.8 Gy to pelvis
RTOG 9413 (1995-1999)	Prostate-only RT + adjuvant ADT<br>WPRT + adjuvant ADT<br>Prostate-only RT + neoadjuvant/concurrent ADT<br>WPRT + neoadjuvant/concurrent ADT	PSA≤100<br>Ineligible for RTOG 9408	Progression free survival	70.2 Gy to prostate<br>50.4 Gy to pelvis
RTOG 9902 (2000-2004)	RT + 24 mos ADT<br>RT + 24mos ADT+TEEx4	PSA 20-100, GS≥7, any T<br>OR<br>≥cT2, GS 8-10, PSA≤100	Disease-free survival	70.2 Gy to prostate<br>46.8 Gy to pelvis
RTOG 9910 (2000-2004)	RT + 4 mos ADT<br>RT + 9 mos ADT	cT1b-4, PSA>10 but ≤100, and GS 2-6<br>OR<br>cT1b-4, PSA<20, GS 7<br>OR<br>cT1b-c, PSA<20, GS 8-10	Disease-specific survival	70.2 Gy to prostate<br>46.8 Gy to pelvis (if no staging surgery)
RTOG 0126 (2002-2008)	Moderate dose RT<br>High dose RT	cT1b-T2b, GS 2-6, PSA≥10 but <20<br>OR<br>cT1b-T2b, GS 7, PSA<15	Overall survival	70.2 Gy to prostate<br>79.2 Gy to prostate
RTOG 0415 (2006-2009)	Conventionally-fractionated RT<br>Hypofractionated RT	cT1b-T2c, GS 2-6, PSA<10	Disease-free survival	73.8 Gy to prostate<br>70 Gy (in 28 fractions) to prostate

*Patients with cN+ or pN+ disease were not included in our analysis.

ADT, androgen deprivation therapy; CT, computed tomography; GS, Gleason score; PSA, prostate-specific antigen; LHRH, luteinizing hormone releasing hormone; MRI, magnetic resonance imaging; Gy, gray; RT, radiation therapy; TEE, paclitaxel, estramustine, and oral etoposide; WPRT, whole pelvis radiation therapy

© 2021 Ma TM et al. *JAMA Network Open.*

eTable 2. Treatment Strategies As Defined by Individual Trial Treatment Arms

Treatment Strategy	Trial Arms
**RT Alone**	RTOG 9408 RT alone
	RTOG 0126 RT alone (control arm)
	RTOG 0415 Conventionally fractionated RT alone
	RTOG 0415 Moderately hypofractionated RT alone
**RT+STADT**	RTOG 9202 RT + 4 months ADT
	RTOG 9408 RT + 4 months ADT
	RTOG 9413 Prostate-only RT + adjuvant 4 months ADT
	RTOG 9413 Whole pelvis RT + adjuvant 4 months ADT
	RTOG 9413 Prostate-only RT + neoadjuvant/concurrent 4 months ADT
	RTOG 9413 Whole pelvis RT + neoadjuvant/concurrent 4 months ADT
	RTOG 9910 RT + 4 months ADT<br>RTOG 9910 RT + 9 months ADT
**RT+LTADT**	RTOG 9202 RT + 28 months ADT
	RTOG 9902 RT + 24 months ADT
	RTOG 9902 RT + 24 months ADT + TEEx4
**High Dose RT**	RTOG 0126 high dose RT (experimental arm)

ADT, androgen deprivation therapy; LTADT, long-term ADT; RT, radiation therapy; STADT, short-term ADT; TEE, paclitaxel, estramustine, and oral etoposide

© 2021 Ma TM et al. *JAMA Network Open.*

eTable 3. Crude Event Rates Per Trial, Stratified by Race and Treatment Strategy

		Black					White				
			Number of Events					Number of Events			
**Trial**	**Treatment**	**Total**	**BCR**	**DM**	**PCSM**	**ACM**	**Total**	**BCR**	**DM**	**PCSM**	**ACM**
RTOG 9202	RT+STADT	83	43	19	13	62	617	375	159	115	501
RTOG 9202	RT+LTADT	103	47	13	8	81	611	269	106	83	482
RTOG 9408	RT Alone	197	84	17	17	135	754	375	80	95	521
RTOG 9408	RT+STADT	197	52	11	10	113	743	280	53	51	516
RTOG 9413	RT+STADT	322	150	49	0	220	881	443	193	0	667
RTOG 9902	RT+LTADT	102	51	6	0	33	265	149	43	0	105
RTOG 9910	RT+STADT	246	57	6	0	70	1190	308	71	0	371
RTOG 0126	RT Alone	84	26	3	0	19	643	300	45	17	192
RTOG 0126	High Dose	104	30	6	2	27	609	186	24	4	177
RTOG 0415	RT Alone	192	16	0	0	13	871	69	0	3	85
**Total**		1630	556	130	50	773	7184	2754	774	368	3617
**Crude Event Rate**			**34.1%**	**8%**	**3.1%**	**47.4%**		**38.3%**	**10.8%**	**5.1%**	**50.3%**

ACM, all-cause mortality; BCR, biochemical recurrence; DM, distant metastasis; LTADT, long-term androgen deprivation therapy; PCSM, prostate cancer-specific mortality; RT, radiotherapy; STADT, short-term androgen deprivation therapy

*Based on trial outcomes, patients enrolled on RTOG 9413, 9902, and 9910 were considered to have received the same treatment in all arms.

© 2021 Ma TM et al. *JAMA Network Open.*
